# Alkalizing Reactions Streamline Cellular Metabolism in Acidogenic Microorganisms

**DOI:** 10.1371/journal.pone.0015520

**Published:** 2010-11-30

**Authors:** Stefania Arioli, Enzio Ragg, Leonardo Scaglioni, Dimitrios Fessas, Marco Signorelli, Matti Karp, Daniele Daffonchio, Ivano De Noni, Laura Mulas, Marco Oggioni, Simone Guglielmetti, Diego Mora

**Affiliations:** 1 Dipartimento di Scienze e Tecnologie Alimentari e Microbiologiche, Università degli Studi di Milano, Milan, Italy; 2 Dipartimento di Scienze Molecolari Agroalimentari, Università degli Studi di Milano, Milan, Italy; 3 Department of Chemistry and Bioengineering, Tampere University of Technology, Tampere, Finland; 4 Laboratorio di Microbiologia Molecolare e Biotecnologia, Dipartimento Biologia Molecolare, Università di Siena, Siena, Italy; Auburn University, United States of America

## Abstract

An understanding of the integrated relationships among the principal cellular functions that govern the bioenergetic reactions of an organism is necessary to determine how cells remain viable and optimise their fitness in the environment. Urease is a complex enzyme that catalyzes the hydrolysis of urea to ammonia and carbonic acid. While the induction of urease activity by several microorganisms has been predominantly considered a stress-response that is initiated to generate a nitrogen source in response to a low environmental pH, here we demonstrate a new role of urease in the optimisation of cellular bioenergetics. We show that urea hydrolysis increases the catabolic efficiency of *Streptococcus thermophilus*, a lactic acid bacterium that is widely used in the industrial manufacture of dairy products. By modulating the intracellular pH and thereby increasing the activity of β-galactosidase, glycolytic enzymes and lactate dehydrogenase, urease increases the overall change in enthalpy generated by the bioenergetic reactions. A cooperative altruistic behaviour of urease-positive microorganisms on the urease-negative microorganisms within the same environment was also observed. The physiological role of a single enzymatic activity demonstrates a novel and unexpected view of the non-transcriptional regulatory mechanisms that govern the bioenergetics of a bacterial cell, highlighting a new role for cytosol-alkalizing biochemical pathways in acidogenic microorganisms.

## Introduction

The mechanism that acts in the regulation of cell bioenergetics belongs to the complexity of biological systems in which large networks of metabolic pathways interact to govern the life and responsiveness of cells in a very significant fashion. Biological systems are constituted by a large number of components and processes whose complex relationships determine properties like dynamics, regulation, and adaptation [1]. In this context, the urease of the dairy bacterium *Streptococcus thermophilus* represents an interesting example of a multifunctional enzymes involved in several metabolic processes. Urease is a complex enzyme that is encoded by an 11-gene operon that accounts for 0.9% of the estimated core genome of *Streptococcus thermophilus* [2], [3]. This enzyme has been found in all previously characterised *S. thermophilus* strains, and urease-negative mutants are not common in nature [4]. Although the physiological role of the *S. thermophilus* urease has not been completely assessed, it was found to be involved in the nitrogen metabolism by a mechanism sensitive to aspartate, glutamate, glutamine and ammonia concentration [5], [6]. The role of urease as a stress response to counteract the acidic challenge, as it is described in several microorganisms [7], is unlike to be applicable in *S. thermophilus* because urea degradation occurs at a relatively high pH that is not associated with a significant loss of viability [8]. The *S. thermophilus* genome has mainly evolved following divergent evolution from the phylogenetically related pathogenic streptococci. Loss-of-function mutations, counterbalanced by the acquisition of relevant traits [e.g. lactose utilisation] have resulted in a *S. thermophilus* genome that is well-adapted for dairy colonization [3], [9]. Because urease is not common in pathogenic streptococci [10], its acquisition and maintenance within the *S. thermophilus* genome is likely dependent upon its contribution to the environmental fitness of this microorganism. The use of a multitechnique, metabolomic approach has highlighted a “hidden” physiological role of urease enzymatic activity in *S. thermophilus*.

## Results

### Urea-stimulated ATP synthesis

**Figure 1:**
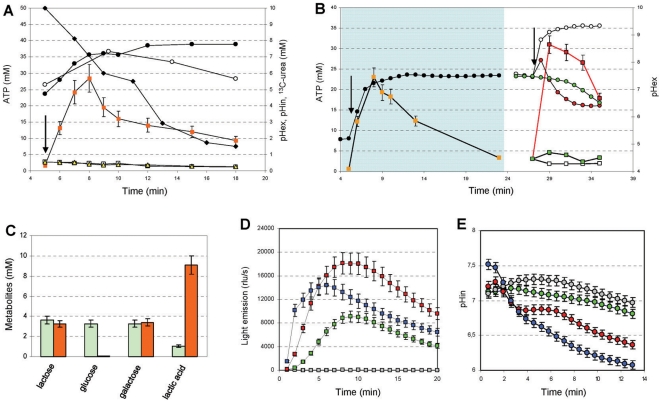
Effects of urea hydrolysis on cellular ATP concentration and homolactic fermentation. A: Changes in the extracellular pH (pH_ex_) (filled circles), intracellular pH (pH_in_) (open circles), intracellular ATP concentration (squares), and ^13^C-urea concentration (diamonds) in a suspension of wild-type *S. thermophilus* cells without (filled squares) or with (white squares) 10 µM of the urease inhibitor flurofamide during urea hydrolysis. The intracellular ATP concentration was also evaluated in the urease-negative mutant A16(*ΔureC3*) (triangles). Shaded panel in B: Changes in pH_ex_ (circles) and intracellular ATP concentration (squares) during the preparation of energetically discharged cells (EdC). White panel in B: changes in the pH_ex_ (circles) and the intracellular ATP concentration (squares) in EdC in the presence of either 10 mM urea (white symbols), 28 mM lactose (green symbols) or both lactose and urea (red symbols). In A and B the addition of urea and/or lactose is indicated by the arrows. C: the consumption of lactose and production of glucose, galactose, and lactic acid in EdC that were activated with lactose (green bars) or lactose and urea (orange bars) under the experimental conditions described in b (white panel). D: the intracellular ATP concentration (reported as light emission) and the intracellular pH, E. MIM945 EdC were activated with 14 mM lactose (green symbols), 14 mM lactose and 0.5 mM urea (red symbols), 14 mM lactose and 1 mM ammonia (blue symbols) or 14 mM lactose and 0.5 mM urea and 0.4 mM sodium oxamate (grey symbols). All of the experiments in this panel were performed in the presence of 100 µg/ml of chloramphenicol to block the protein synthesis. The error bars represent the SEM.

In several bacterial species, such as *Ureaplasma urealyticum* [11], *Bacillus pasteurii* [12], *Helicobacter pylori* [13] and *Howardella ureilytica* [14], urea hydrolysis generates an ammonium/urea-dependent chemical potential that is coupled to ATP synthesis. The addition of 10 mM urea to a suspension of nutrient-starved *S. thermophilus* cells resulted in a rapid increase in the intracellular ATP concentration and a concomitant extracellular and intracellular alkalisation that was caused by increased urease activity (Figure 1A). In the presence of the urease inhibitor flurofamide the intracellular ATP concentration did not increase. While the extracellular pH (pH_ex_) remained relatively alkaline, the intracellular pH (pH_in_) became acidic, and this change was not dependent upon urease activity, which persisted until approximately 83% of the urea molecules were consumed. The urea-stimulated ATP synthesis was not based on a chemiosmotic mechanism, since protonophore, ionophore or ATPase inhibitors did not reduce ATP synthesis (Table S1), which was also detected in a membrane-free cell extract (Figure S1).

### Urea hydrolysis streamlines cellular metabolism

Since the metabolism of *S. thermophilus* is exclusively based on the homolactic fermentation of lactose via the Embden-Meyerhof glycolytic pathway [15], we hypothesized that urea hydrolysis increases the pH_in_ and optimizes the activity of the glycolytic enzymes, thereby increasing the rate of ATP synthesis. An increase in the rate of ATP synthesis was detected in the absence of carbon sources (Figure 1A), suggesting that high levels of glycolytic intermediates were still present in the cells that were collected at the beginning of the stationary growth phase. To corroborate these hypotheses, the change in ATP concentration in the presence of urea, lactose or a mixture of the two compounds was studied in energetically discharged cells (EdC) prepared as described in Materials and Methods S1. Urea hydrolysis effectively increased the intracellular ATP pool (Figure 1B, C and D) only when the EdC were activated with lactose (red line in Figure 1B). An increase in the pH_ex_ confirmed that urease was still active in the EdC. When EdC were provided with urea and lactose, the extracellular pH increased, and there was a tenfold increase in the intracellular ATP concentration, which was followed by rapid acidification (Figure 1B) and lactic acid production (Figure 1C). While urea did not affect the rate of lactose consumption by EdC, it did cause a significant increase in lactic acid production (Figure 1C). The high glucose concentration that was measured in lactose-activated EdC (Figure 1C) indicates that in the absence of urea hydrolysis, the production rate of glucose by *S. thermophilus* was higher than the rate of glucose consumption; therefore, the glycolytic pathway represents the rate-limiting step of metabolism.

To explore this phenomenon, we measured the intracellular ATP concentration in the presence of D-luciferin via light emission by the bioluminescent strain MIM945, which is a derivative of *S. thermophilus* DSM20617^T^ [16]. The light emission of the MIM945 EdC was significantly higher when the bacteria were supplemented with lactose and urea or lactose and ammonia, compared to lactose alone (Figure 1D). Furthermore, the inhibition of light emission by sodium oxamate, a glycolytic inhibitor [17] (Figure 1D), confirmed that the urea/ammonia-dependent ATP synthesis, and the related light emission, were linked to glycolytic enzymes activity. Sodium oxamate is an analogue of pyruvate which blocks glycolysis by competitively inhibiting lactate dehydrogenase [17]. The activation of metabolism in the EdC by urea and ammonia indicates that urea hydrolysis accelerates the glycolytic flux and homolactic fermentation by an intracellular alkalisation that is generated by the release of ammonia into the cytoplasm.

The evaluation of the intracellular pH variation during the activation of the metabolism in EdC revealed an apparent paradox: the highest decrease in intracellular pH was obtained in presence of alkalizing molecules such as urea and ammonia. Following EdC activation with lactose/urea or lactose/ammonia, the intracellular acidification rate was increased compared to activation with lactose alone (Figure 1E). The cytoplasm alkalization generated by urea hydrolysis, masked by the buffering effect of the lactic acid produced by the homolactic fermentation, was highlighted by treating the MIM945 EdC with the glycolysis inhibitor sodium oxamate (Figure 1E).

**Figure 2:**
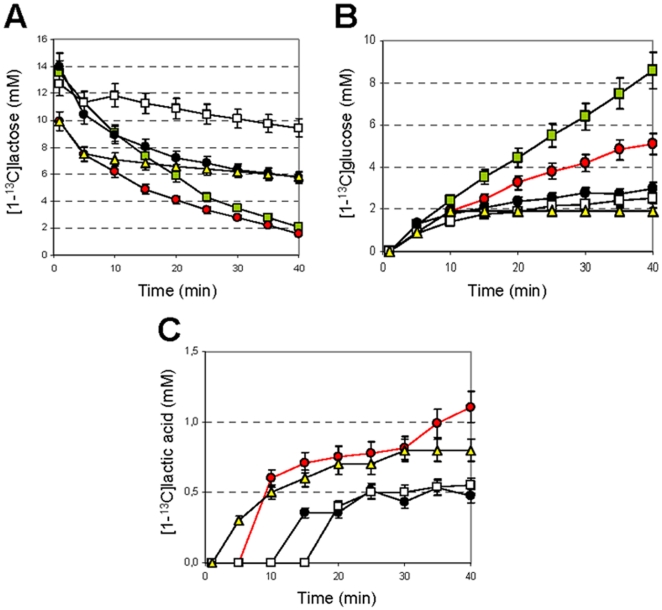
Dynamics of metabolite pools in *S. thermophilus* as determined by in vivo NMR. Time course for (1-^13^C)lactose (14 mM) (A), -glucose (B), and -lactic acid (*C*), consumption/product formation in wild-type *S. thermophilus*. The metabolite concentrations were measured in *in vivo* ^13^C NMR experiments using EdC that were activated with 14 mM lactose (green symbols), 14 mM lactose and 0.5 mM urea (black symbols), 14 mM lactose and 10 mM urea (red symbols), 14 mM lactose and 1 mM ammonia (yellow symbols) or 14 mM lactose, 0.5 mM urea and 0.4 M sodium oxamate (white symbols). The (1-^13^C)-lactic acid concentration in EdC that were activated with 14 mM lactose was always below the detection limit using the instrument parameters that are listed in Materials and Methods S1. The error bars represent the SEM.

The stimulation of *S. thermophilus* metabolism by urea hydrolysis and ammonia was confirmed using *in vivo* NMR analysis of EdC that were activated with (1-^13^C)-lactose. The dynamics of the lactose, glucose and lactic acid concentrations (Figure 2) show that the presence of urea and ammonia strongly affected the rate of lactose consumption and the increase in lactic acid production, which remained below the detection limit when the EdC were supplied with (1-^13^C)-lactose only (Figure 2C). Similarly, in the absence of urea or ammonia, the glucose concentration increased to 8.6 mM, confirming that glycolysis is the rate-limiting step of metabolism in *S. thermophilus* (Figure 2B).

Analogous experiments that were performed using *L. lactis* IL1403 treated with (1-^13^C)-glucose with and without 1 mM ammonia, showed that intracellular alkalization also caused increased lactic acid production in this species (Figure S2).

**Figure 3:**
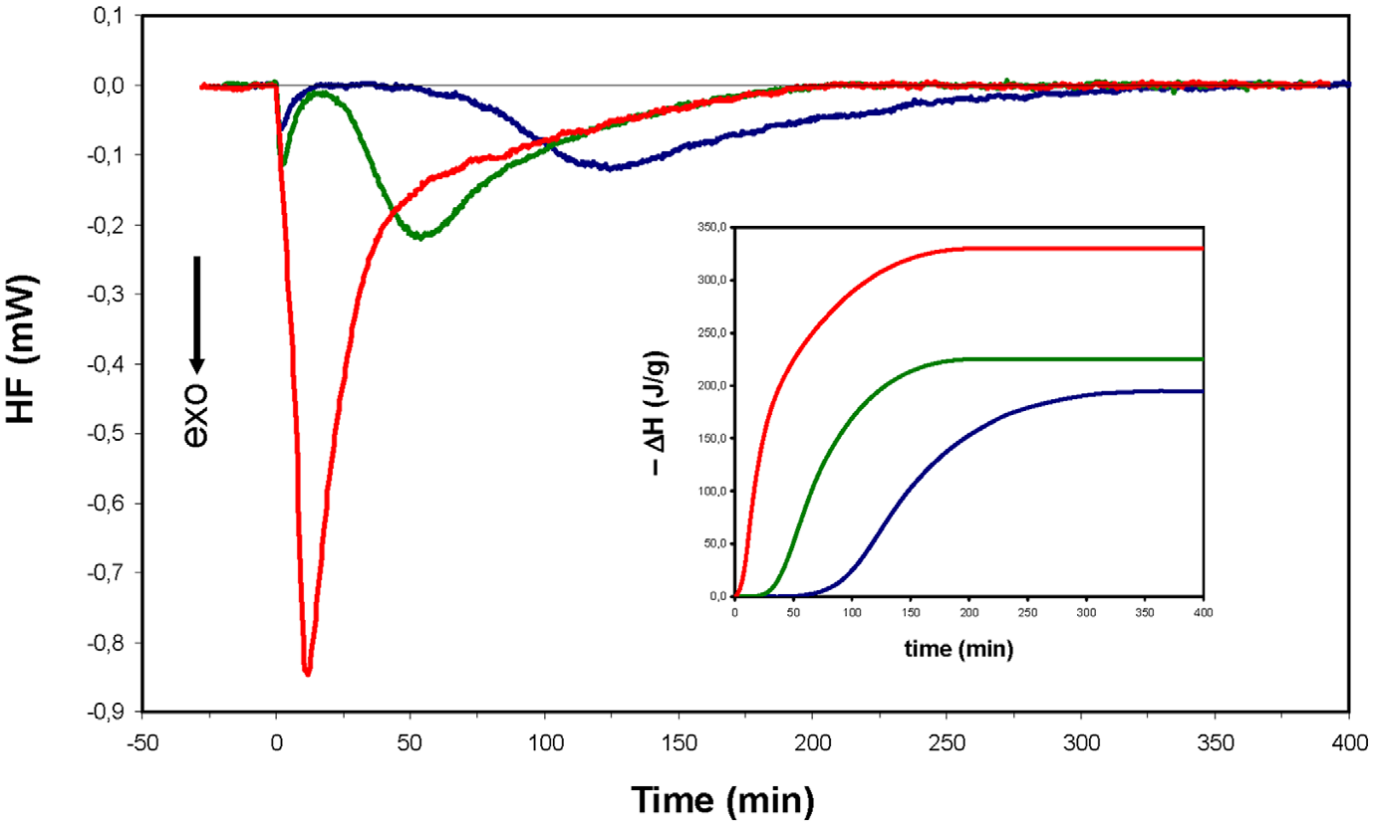
Raw isothermal titration calorimetry data (heat flux versus time) of *Streptococcus thermophilus* lactose metabolism alone (blue line) or in the presence of ammonia (green line) or urea (red line). Lactose (70 µmol), ammonia (5 µmol) or urea (2.5 µmol), was injected into a 5 ml suspension of EdC at time zero. The inset represents the overall specific enthalpy (with respect to grams of total protein) versus time. The details of the experimental conditions are provided in the supplementary materials.

Since cell metabolism is accompanied by enthalpy change, heat dissipation measured by calorimetry represents a suitable procedure to monitor metabolic activity. Based on calorimetric experiments, the specific change in enthalpy (ΔH per g of protein) in *S. thermophilus* increased 70% and 15% in the presence of lactose/urea and lactose/ammonia respectively, compared to the heat that was released by cells that were fermenting lactose only (Figure 3). Similar data were obtained employing *L. lactis* cells that were given ammonia as an alkalizing agent (Figure S3).

### Urea hydrolysis has a cooperative behaviour in a mixed bacterial community

**Figure 4:**
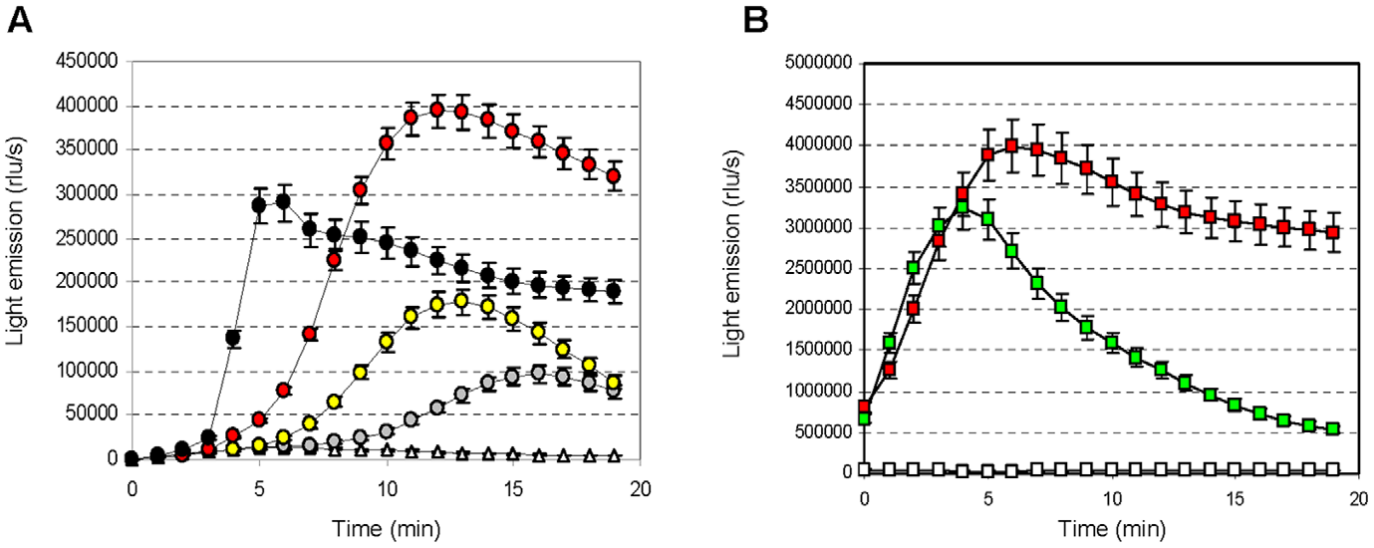
The cooperative and altruistic behaviour of *S. thermophilus* urease activity in mixed bacterial communities. (A) The intracellular ATP concentration presented as light emission, in *S. thermophilus* A16-945 (urease-negative) EdC in mixed cultures. The A16-945 EdC were mixed with the wild-type EdC at the following ratios (v/v): 100% A16-945 (white triangles), 95% A16-945 (grey circles), 90% A16-945 (yellow circles), 80% A16-945 (red circles), and 50% A16-945 (black circles). The EdC were activated with 14 mM lactose/0.5 mM urea. (B) The intracellular ATP concentration, presented as light emission, in *Lactococcus lactis* 1403-945 (urease-negative) EdC in mixed cultures. The urease-negative EdC were mixed with the wild-type EdC at a 1∶1 ratio. The mixed EdC cultures were acitvated with 0.5 mM urea (white squares), 14 mM lactose (green squares) or 14 mM lactose/0.5 mM urea (red squares). The error bars represent the SEM.

Urea hydrolysis determines the release of ammonia into the medium varying the pH towards alkaline values. Does the medium alkalinization by urease-positive cells has an effect on the cell bioenergetics of urease-negative microorganisms sharing the same environment? To answer this question mixed-strains or –bacteria suspensions of the urease-positive *S. thermophilus* wild-type DSM20617^T^ strain, the luminescent urease-negative A16-945 strain (a *ΔureC3* derivative of strain DSM20617^T^) [2] and the luminescent urease-negative *Lactococcus lactis* 1403-945 strain (a derivative of strain IL1403) were analyzed by measuring the light emission in presence of lactose or lactose and urea. The results obtained clearly indicated that urea hydrolysis of the wild-type *S. thermophilus* mixed-strain cultures coincided with a significant increase in the light emission from the urease-negative cells, indicating that there was an improvement in the energy flux of the urease-negative strains. This result revealed a cooperative and altruistic beneficial effect [18], [19] of urease activity in mixed microbe communities (Figure 4).

### *S. thermophilus* metabolism is optimized to work at alkaline pH

**Figure 5:**
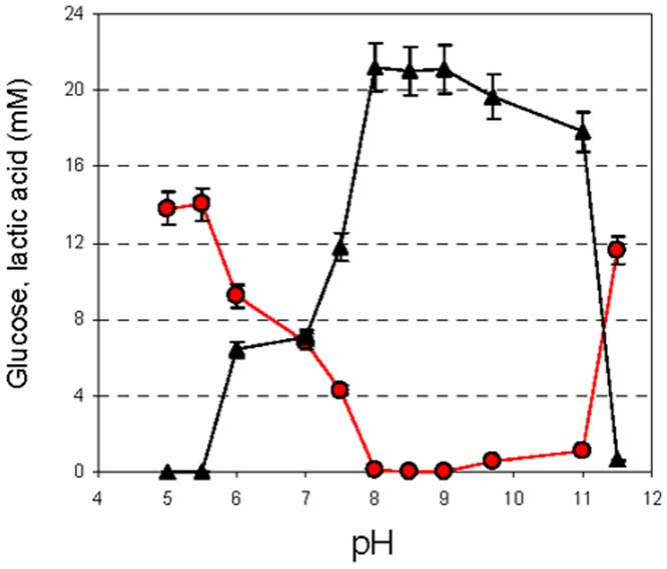
pH-dependent glucose consumption (red circles) and lactic acid production (black triangles) in EdC of *S. thermophilus* that were treated with 100 µM of the uncoupler gramicidine. The error bars represent the SEM.

**Figure 6:**
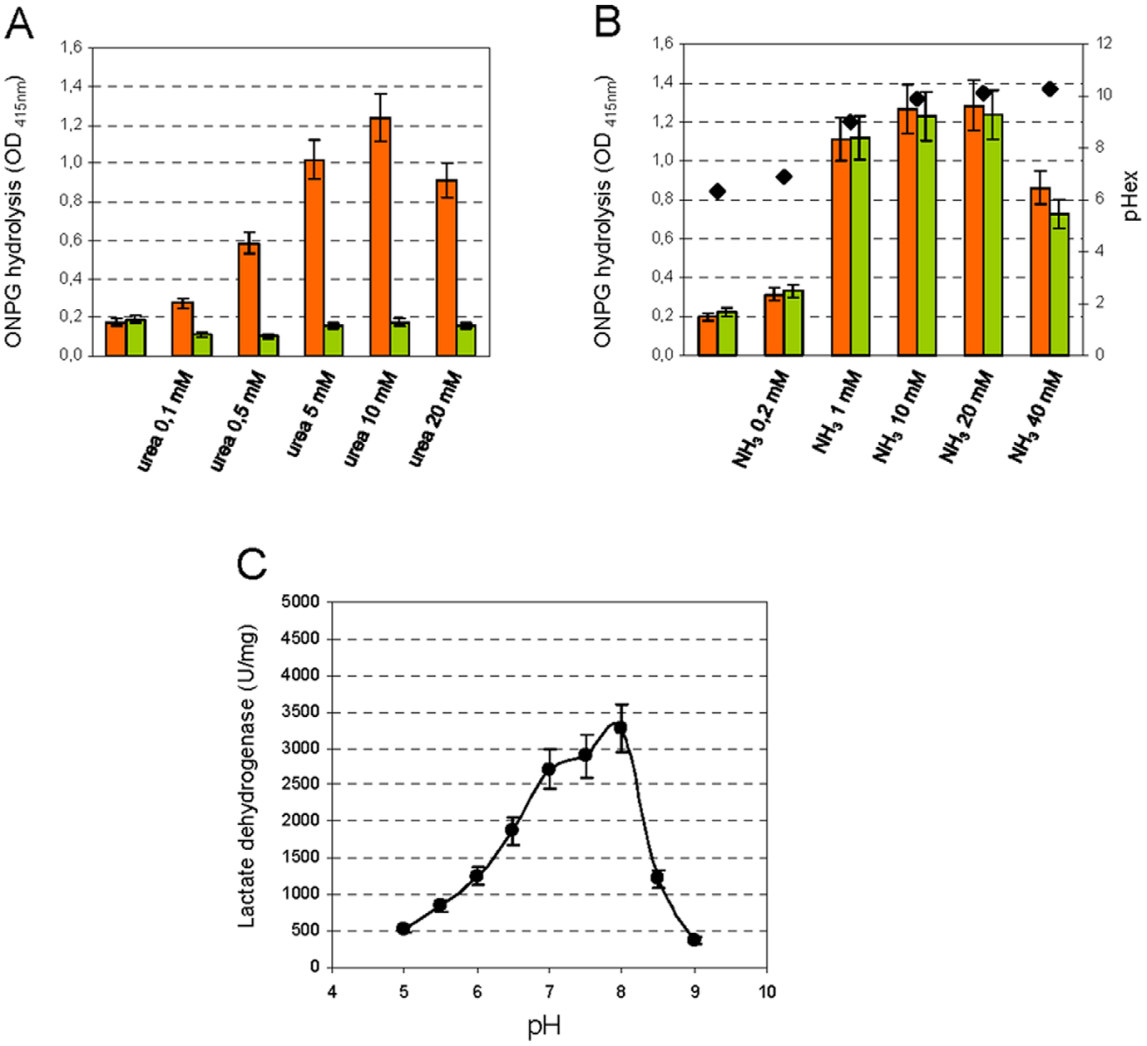
Urea-dependent β-galactosidase activity and pH-dependent lactate dehydrogenase activity. The dependence of β-galactosidase activity on the urea (A) and ammonia concentration (B) in wild-type *S. thermophilus* wild-type (orange bars) and urease-negative A16(*ΔureC3*) (green bars) permeabilised cell suspensions. The extracellular pH (♦) is indicated. (B). The pH-dependent lactate dehydrogenase activity (C) was measured in crude cell extracts. All of the experiments in this panel were performed in presence of 100 µg/ml of chloramphenicol to block the protein synthesis. The error bars represent the SEM.

*S. thermophilus* cell suspensions were treated with the protonophore uncoupler gramicidine to study the effect of pH on glycolytic enzyme activity. Gramicidine is a protonophore carrier that promote transmembrane movement of protons allowing the equilibration between pH_ex_ and pH_in_. By adjusting the pH_ex_ values, and therefore the pH_in_, it was possible to evaluate the glycolysis and the homolactic fermentation efficiency by measuring the glucose consumption and the lactic acid production in a defined time period. The glycolytic enzymes had maximum activity at alkaline pH values and the maximum glucose consumption was observed between pH 8 and 11 (Figure 5). Based on the glucose consumption measurements, the maximum production of lactic acid occurred between pH 8 and 9 β-galactosidase and lactate dehydrogenase, the two enzymes that are upstream and downstream respectively, in the glycolytic pathway, had optimum activity at alkaline pH values (Figure 6) [20], [21]. Interestingly, the β-galactosidase activity was strongly influenced by urea hydrolysis, and an increase in its activity was dependent upon the urea or ammonia concentration (Figure 6A and B). Because it was demonstrated that β-galactosidase increases the efficiency of transport by hydrolyzing lactose and making galactose available for the exchange reaction via the lactose permease LacS [22], we could speculate that urea hydrolysis increases lactose intake in *S. thermophilus* cells. This hypothesis was corroborated by the time course of (1-^13^C)-lactose consumption in EdC activated with lactose without or with 10 mM urea (Figure 2A).

### Urea-mediated alkalisation is a non-transcriptional mechanism to regulate cellular bioenergetics

All the previous experiments were carried out in presence of chloramphenicol to avoid the interference of newly synthesized proteins on the metabolic parameters measured during urea hydrolysis or the cytoplasm alkalization induced by ammonia. Here we evaluated the effect of cytoplasm alkalization on the transcription of six different genes that encode enzymes directly involved in the cellular bioenergetic machinery (*lacS,* lactose permease; *lacZ,* β-galactosidase; *gapA1*, glyceraldehyde-3-phosphatedehydrogenase; *pgk*, phosphoglycerate kinase; *pyk*, pyruvate kinase; *ldh*, lactate dehydrogenase). The experiments, performed in absence of chloramphenicol, did not reveal significant change in the transcript level of any of these genes in the presence of urea or other alkalizing agents (Figure S4). This data allowed to conclude that, in *S. thermophilus*, the modulation of the intracellular pH toward alkaline values, controlled by urea hydrolysis and not a transcriptional regulation, represents the main regulatory mechanism of cellular bioenergetics.

## Discussion

Fermentation processes involve the oxidation of carbohydrates to generate a range of products including weak organic acids. Weak acids, such as lactic acid, have potent antimicrobial activity because their undissociated form pass freely through the cell membrane leading to the acidification of cytoplasm. Until now, the acid resistance mechanisms have been investigated to clarify how acidogenic bacteria can survive the by products of their own metabolism and to understand the responses available to spoilage and pathogenic organisms in low-pH environments [7]. The effect of acid resistance mechanisms on cell bioenergetics has not yet been experimentally investigated even if it is well established the relatively acid-sensitivity of glycolytic enzymes [7]. Here we show that urease is not a stress-response to a low environmental pH and based on the data presented in this study, we propose the following physiologic model of urease activity in *S. thermophilus*. Urease activity which is stimulated when the milk pH is weakly acidic (pH 5.8–6) [3], [23], should be considered a regulatory system that has evolved to optimize the activity of the glycolytic enzymes. These enzymes are exposed to an increasingly acidic intracellular environment and must maintain the cell energy homeostasis when the pH_ex_ and pH_in_ decrease as a result of lactic acid production. Urease biogenesis [23] is only important when the cells are actively growing, since it increases the fermentative capacity of *S. thermophilus* and leads to rapid growth [15] and an increased acidification rate in milk. Since the activity of the bioenergetic machinery modulates the intracellular pH, the mechanism of metabolism regulation in other urease-positive bacteria, including human pathogens, should be further analyzed. All of the metabolic reactions that results in alkalisation of the cytosol of acidogenic organisms, such as those involved in the arginine deiminase (ADI) pathway, the citrate metabolism or those involved in the malolactic conversion [7]; [24]; [25] should be analysed in light of these novel findings. The conserved role of alkalizing reactions across acidogenic bacteria is also supported by the data obtained using the bioluminescents EdC of *Lactococcus lactis* IL1403 (ADI positive) and *S. pneumoniae* SP292-945 (ADI-positive) activated with glucose or cellobiose supplemented with ammonia (Figure S5).

The modulation of *S. thermophilus* metabolism by urease activity provides a novel perspective on the complexity of the regulatory mechanisms that governs cell biology [1]. The mechanism presented here is a novel example of how the metabolism of microorganisms has evolved in response to a defined environment. It has been recently proposed that the highly efficient fermentative capacity of the baker's yeast *Saccharomyces cerevisiae* is a results of a whole-genome duplication event that occurred during its speciation in environmental habitat where glucose resources became both large and dense [26]. According to another strategy, the acquisition of urease operon by *S. thermophilus* confers a metabolic advantage because the proteins encoded by this operon increase the fermentative capacity in a lactose-rich environment that contains urea, such as milk.

## Materials and Methods

### Bacterial strains and mutants

The bioluminescent derivatives mutants of *S. thermophilus* DSM 20617^T^, the urease-negative mutant A16(*ΔureC3*) [2] and *Lactococcus lactis* IL1403, were created by electrotransformation with the shuttle vector pCSS945, which contains the luciferase gene from *Pyrophorus plagiophtalamus* [16].

### Laboratory methods

A detailed description of the *in vivo* ^13^C- and ^31^P-NMR NMR spectroscopy is provided in the supplementary materials. The intracellular pH (pH_in_) was estimated using NMR as described by Voit et al. [1] and Neves et al. [27]. The intracellular pH of *S. thermophilus* was measured at external pH values of 6.0 to 9.0 using a pH-sensitive fluorescence probe 5 (and 6-)-carboxyfluorescein succinimidyl ester (CFSE) as described by Sawatari and Yokota [28], which was based on the method originally described by Breeuwer et al. [29]. The experimental protocol for the preparation of energetically discharged *S. thermophilus* and *Lactococcus lactis* cells (EdC) is provided in the supplementary materials. The detailed experimental setup for the measurement of the intracellular ATP concentration, light emission, pH and glycolytic and homolactic fermentation in the EdC is also provided in the supplementary materials. The ATP concentration in whole cells, cell-free extracts and membrane-free extracts was determined as previously described by Jahns [12]. The change in enthalpy measurements were performed using a calorimetric approach that is described in the supplementary materials. The measurement of the pH-dependent glycolysis and homolactic fermentation activities were carried out as described by Nannen and Hutkins [30] using 100 µM gramicidine as the membrane uncoupling agent. Quantitative RT-PCR, as described in the supplementary materials, was used to analysed the transcription of genes that are involved in *S. thermophilus* metabolism. The glucose and lactic acid concentrations were measured using HPLC, and the assays used to determine the enzymatic activity were performed as previously described by Krishnan et al. [31] and Gaspar et al. [32].

## Supporting Information

Table S1 ***S. thermophilus*** **cells were incubated in the presence of the indicated compounds for 5 min at 37°C prior the addition of urea to a final concentration of 10 mM.** The ATP content was determined 8 min later. ^a^  =  values are presented as the mean ± standard deviation. The activity of gramicidine, m-chlorophenylhydrazone (CCCP), N-dicyclohexylcarbodiimide (DCCD) and valinomycin on the overall metabolism of *S. thermophilus* was evaluated by determining the minimal concentration that inhibited the growth of *S. thermophilus* and comparing the data obtained (expressed as mol of the chemical per CFU) with the concentration of the same metabolic inhibitors used for the evaluation of urea-dependent ATP synthesis. Gramicidine inhibited *S. thermophilus* growth at a mol/CFU value of 3×10^-18^ and was used for ATP measurement at a higher mol/CFU value of 4×10^-17^. CCCP, valinomycin and DCCD inhibited *S. thermophilus* growth at a mol/CFU value of 8×10^-15^, 8×10^-17^ and 8×10^-15^, respectively, and were used for ATP measurement at a similar mol/CFU value of 4×10^-15^, 4×10^-17^ and 4×10^-15^, respectively. These data demonstrate that a similar concentration of chemicals was used to inhibit *S. thermophilus* growth and during the evaluation of the intracellular ATP concentration.
(DOC)

Figure S1 **Measurement of the ATP concentration in total cell extracts (Ec) or membrane-free extracts (MfEc) of** ***S. thermophilus*** **after the addition of 10 mM urea (grey bars) supplemented with 10 µM flurofamide (a urease inhibitor; white bars) or 0.1% (v/v) Triton X-100 (dark grey bars).** The black bars represent the ATP concentration measured after the addition of urea to heat-treated (100 °C for 5 min) Ec or MfEc. The errors bars represent SEM.
(TIF)

Figure S2 **Time course of [1-^13^C]-glucose (14 mM).** (**A**) and -lactic acid (**B**) consumption/product formation in *L. lactis* IL1403. The metabolite concentrations were measured in *in vivo* ^13^C NMR experiments using EdC that were activated with 14 mM lactose (white circles) or 14 mM lactose/1 mM ammonia (black circles). The error bars represent the SEM.
(TIF)

Figure S3 **Raw isothermal titration calorimetry data (heat flux versus time) of** ***L. lactis*** **IL1403 lactose metabolism alone (blue line), or in the presence of ammonia (red line).** The effect of ammonia dilution is also shown (green line). Lactose (70 mmol) or ammonia (5 mmol) was injected into a 5 ml suspension of EdC at time zero. The inset represents the overall specific enthalpy (with respect to grams of total protein) versus time. The details of the experimental conditions are provided in the supplementary materials.
(TIF)

Figure S4 **The relative expression of** ***S. thermophilus*** **genes involved in metabolism.** *lacS,* lactose permease; *lacZ,* β-galactosidase; *gapA1*, glyceraldehyde-3-phosphatedehydrogenase; *pgk*, phosphoglycerate kinase; *pyk*, pyruvate kinase; *ldh*, lactate dehydrogenase. Total RNA was extracted from EdC, EdC activated with 14 mM lactose (EdCL), EdC activated with lactose and 1 mM urea (EdCLU), EdC activated with lactose and 1 mM ammonia (EdCLNH3) or EdC treated with NH3 (EdCNH3) or urea (EdCU). The expression levels of analyzed genes was normalized using *polC*, *rpoC* and *murE* as reference housekeeping genes.
(TIF)

Figure S5 **The intracellular ATP concentration presented as light emission, in** ***S. pneumoniae*** **FP292-945 EdC activated with 14 mM glucose (white circles) or 14 mM glucose and 1 mM ammonia (black circles) (A).** The intracellular ATP concentration presented as light emission, in *S. pneumoniae* FP292-945 EdC activated with 14 mM cellobiose (white circles) or 14 mM cellobiose and 1 mM ammonia (black circles) (**B**). The error bars represent the SEM.
(TIF)

Materials and Methods S1 (DOC)

## References

[1] Voit E, Neves AR, Santos H (2006). The intricate side of systems biology.. Proc Natl Acad Sci U S A.

[2] Mora D, Maguin E, Masiero M, Ricci G, Parini C (2004). Characterization of urease genes cluster of Streptococcus thermophilus.. J Appl Microbiol.

[3] Hols P, Hancy F, Fontaine L, Grossiord B, Prozzi D (2005). New insights in the molecular biology and physiology of *Streptococcus thermophilus* revealed by comparative genomics.. FEMS Microbiol Rev.

[4] Monnet C, Pernoud S, Sepulchre A, Fremaux C, Corrieu G (2004). Selection and properties of Streptococcus thermophilus mutants deficient in urease.. J Dairy Sci.

[5] Arioli S, Christophe M, Guglielmetti S, Parini C, De Noni I (2007). Aspartate biosynthesis is essential for the growth of Streptococcus thermophilus in milk, and aspartate availability modulates the level of urease activity.. Appl Environ Microbiol.

[6] Monnet C, Mora D, Corrieu G (2005). Glutamine synthesis is essential for the growth of Streptococcus thermophilus in milk and is linked to the catabolism of urea.. Appl Environ Microbiol.

[7] Cotter PD, Hill C (2003). Surviving the acid test: Responses of Gram-positive bacteria to low pH.. Microbiol Mol Biol Rev.

[8] Bolotin A, Quinquis B, Renault P, Sorokin A, Ehrlich SD (2004). Complete sequence and comparative genome analysis of the dairy bacterium *Streptococcus thermophilus*.. Nature Biotech.

[9] Rasmussen TB, Danielsen M, Valina O, Garrigues C, Johansen E (2008). *Streptococcus thermophilus* Core Genome: Comparative Genome Hybridization Study of 47 Strains.. Appl Environ Microbiol.

[10] Mora D, Monnet C, Daffonchio D (2005). Balancing the loss and acquisition of pathogenic traits in food-associated bacteria.. Microbiology-SGM.

[11] Smith DGE, Russell WC, Ingledew WJ, Thirkell D (1993). Hydolysis of urea by *Ureaplasma urealyticum* generates a transmembrane potential with resultant ATP synthesis.. J Bacteriol.

[12] Jahns T (1996). Ammonium/urea-dependent generation of a proton electrochemical potential and synthesis of ATP in *Bacillus pasteurii.*. J Bacteriol.

[13] Meyer-Rosberg K, Scott RD, Rex D, Melchers K, Sachs G (1996). The effect of environmental pH on the proton motive force of *Helicobacter pylori*.. Gastroenterology.

[14] Cook AR, Riley PW, Murdoch H, Evans PN, McDonald IR (2007). *Howardella ureilytica* gen. nov., sp. nov., a Gram-positive, coccoid-shaped bacterium from a sheep rumen.. Int J Syst Evol Microbiol.

[15] Pastink MI, Teusink B, Hols P, Visser S, de Vos WM (2009). Genome-scale model of *Streptococcus thermophilus* LMG18311 for metabolic comparison of lactic acid bacteria.. Appl Environ Microbiol.

[16] Loimaranta V, Tenovuo J, Koivisto L, Karp M (1998). Generation of bioluminescent *Streptococcus mutans* and its usage in rapid analysis of the efficacy of antimicrobial compounds.. Antimicrob Agents Chemother.

[17] Liu H, Hu YP, Savaraj N, Priebe W, Lampidis TJ (2001). Hypersensitization of tumor cells to glycolytic inhibitors.. Biochem.

[18] Griffin AS, Stuart AW, Buckling A (2004). Cooperation and competition in pathogenic bacteria.. Nature.

[19] Gore J, Youk H, van Oudenaarden A (2009). Snowdrift game dynamics and facultative cheating in yeast.. Nature.

[20] Smart JB, Crow VL, Thomas TD (1985). Lactose hydrolysis in milk and whey using β-galactosidase from *Streptococcus thermophilus*.. New Zealand J Dairy Sci Tech.

[21] Yang ZP, Parkala E, Tupasela T (1993). Lactose hydrolysis by free and fiber-entrapped β-galactosidase from *Streptococcus thermophilus*.. J Agricul Sci.

[22] Geertsma ER, Duurkens RH, Poolman B (2005). The activity of lactose transporter from *Streptococcus thermophilus* is increased by phosphorylated IIA and the action of β-galactosidase.. Biochem.

[23] Mora D, Monnet C, Parini C, Guglielmetti S, Mariani A (2005). Urease biogenesis in *Streptococcus thermophilus*.. Res Microbiol.

[24] Magni C, Mendoza D, Konings WN, Lolkema JS (1999). Mechanism of citrate metabolism in *Lactococcus lactis*: resistance against lactate toxicity at low pH.. J Bacteriol.

[25] Broadbent JR, Larsen RL, Deiel V, Steele JL (2010). Physiological and trabscriptional response of *Lactobacillus casei* ATCC 334 to acid stress.. J Bacteriol.

[26] Conant GC, Wolfe K (2007). Increased glycolytic flux as an outcome whole-genome duplication in yeast.. Mol Syst Biol.

[27] Neves AR, Ramos A, Nunes MC, Kleerebezem M, Hugenholtz J (2004). In vivo nuclear magnetic resonance studies of glycolitic kinetics in *Lactococcus lactis*.. Biotech Bioeng.

[28] Sawatari Y, Yokota A (2007). Diversity and Mechanisms of alkali Tolerance in Lactobacilli.. Appl Environ Microbiol.

[29] Breeuwer P, Drocourt JL, Rombouts FM, Abee T (1996). A novel method for continuous determination of the intracellular pH in bacteria with the internally conjugated fluorescent probe 5 (and 6-)-carboxyfluorescein succinimidyl ester.. Appl Environ Microbiol.

[30] Nannen NL, Hutkins RW (1990). Intracellular pH effects in lactic acid bacteria.. *J Dairy Sci*.

[31] Krishnan S, Gowda LR, Karanth NG (2000). Studies on lactate dehydrogenase of *Lactobacillus plantarum* spp. involved in lactic acid biosynthesis using permeabilizaed cells.. Proc Biochem.

[32] Gaspar P, Neves AR, Shearman CA, Gasson MJ, Baptista AM (2007). The lactate dehydrogenases encoded by the ldh and ldhB genes in *Lactococcus lactis* exibit distinct regulation and catalytic properties – comparative modelling to probe the molecular basis.. FEBS J.

