# Supplementary material for: Alkalizing Reactions Streamline Cellular Metabolism in Acidogenic Microorganisms
Source: PLoS One. 2010 Nov 30;5(11):e15520. doi: 10.1371/journal.pone.0015520 (PMC2994868; doi:10.1371/journal.pone.0015520)
Supplement: Table S1 — S. thermophilus cells were incubated in the presence of the indicated compounds for 5 min at 37°C prior the addition of urea to a final concentration of 10 mM. The ATP content was determined 8 min later. a = values are presented as the mean ± standard deviation. The activity of gramicidine, m-chlorophenylhydrazone (CCCP), N-dicyclohexylcarbodiimide (DCCD) and valinomycin on the overall metabolism of S. thermophilus was evaluated by determining the minimal concentration that inhibited the growth of S. thermophilus and comparing the data obtained (expressed as mol of the chemical per CFU) with the concentration of the same metabolic inhibitors used for the evaluation of urea-dependent ATP synthesis. Gramicidine inhibited S. thermophilus growth at a mol/CFU value of 3×10^-18^ and was used for ATP measurement at a higher mol/CFU value of 4×10^-17^. CCCP, valinomycin and DCCD inhibited S. thermophilus growth at a mol/CFU value of 8×10^-15^, 8×10^-17^ and 8×10^-15^, respectively, and were used for ATP measurement at a similar mol/CFU value of 4×10^-15^, 4×10^-17^ and 4×10^-15^, respectively. These data demonstrate that a similar concentration of chemicals was used to inhibit S. thermophilus growth and during the evaluation of the intracellular ATP concentration. (DOC) [file pone.0015520.s001.doc]

**Table S1.** Effect of metabolic inhibitors on urea-stimulated ATP synthesis in *S. thermophilus*

| Compound(s) | Intracellular ATP (mM) ^a^ |
| --- | --- |
| None | 5.9 ± 0.6 |
| Gramicidine (20 μM) | 6.1 ± 0.5 |
| CCCP (200 μM) | 7.7 ± 0.9 |
| DCCD (200 μM) | 8.6 ± 0.8 |
| Valinomycin (2 μM) and KCl (20 mM) | 8.4 ± 0.7 |
