# Supplementary figures and images for: Alkalizing Reactions Streamline Cellular Metabolism in Acidogenic Microorganisms
Source: PLoS One. 2010 Nov 30;5(11):e15520. doi: 10.1371/journal.pone.0015520 (PMC2994868; doi:10.1371/journal.pone.0015520)

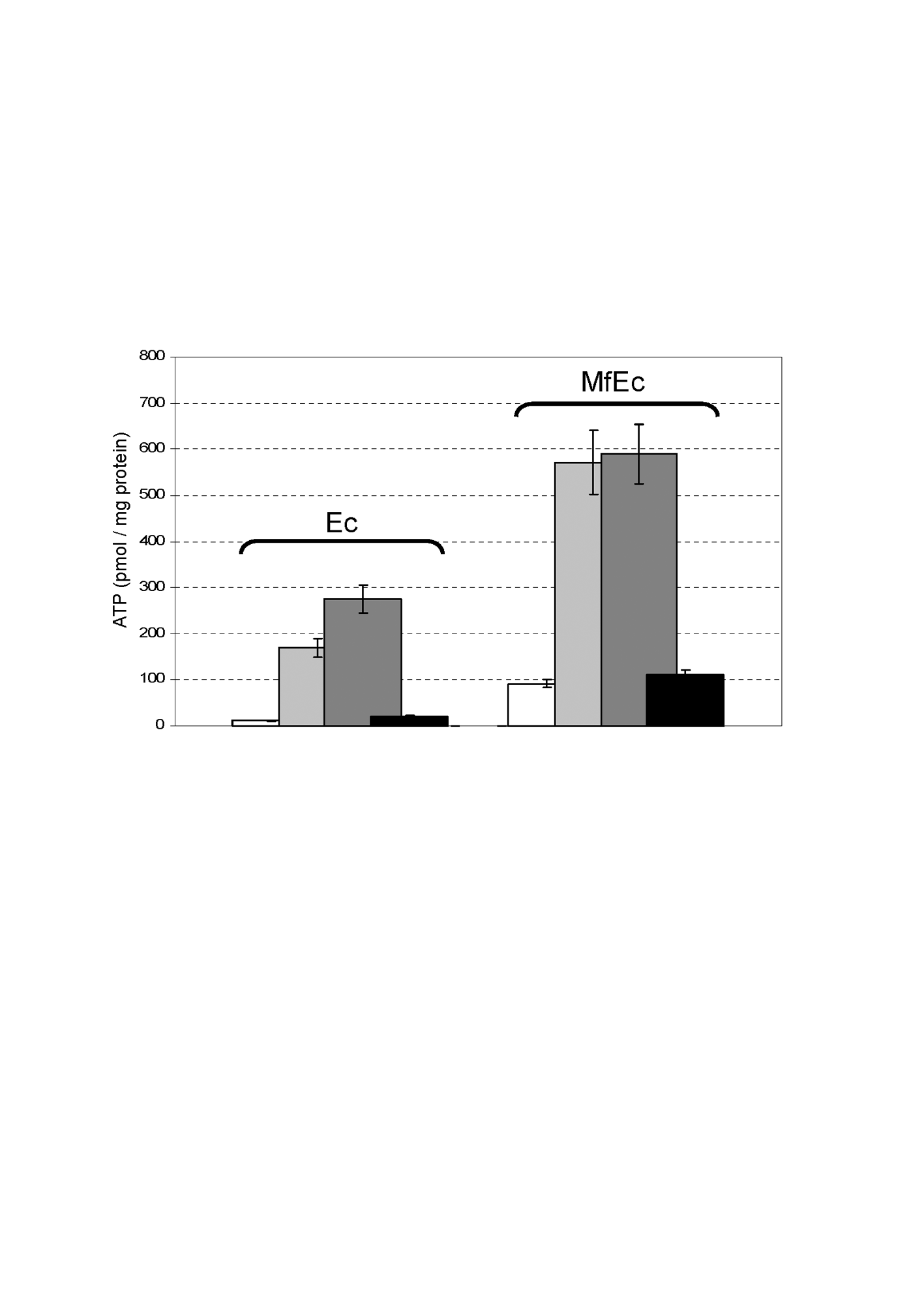

Supplement: Figure S1 — Measurement of the ATP concentration in total cell extracts (Ec) or membrane-free extracts (MfEc) of S. thermophilus after the addition of 10 mM urea (grey bars) supplemented with 10 µM flurofamide (a urease inhibitor; white bars) or 0.1% (v/v) Triton X-100 (dark grey bars). The black bars represent the ATP concentration measured after the addition of urea to heat-treated (100 °C for 5 min) Ec or MfEc. The errors bars represent SEM. (TIF) [file pone.0015520.s002.tif]

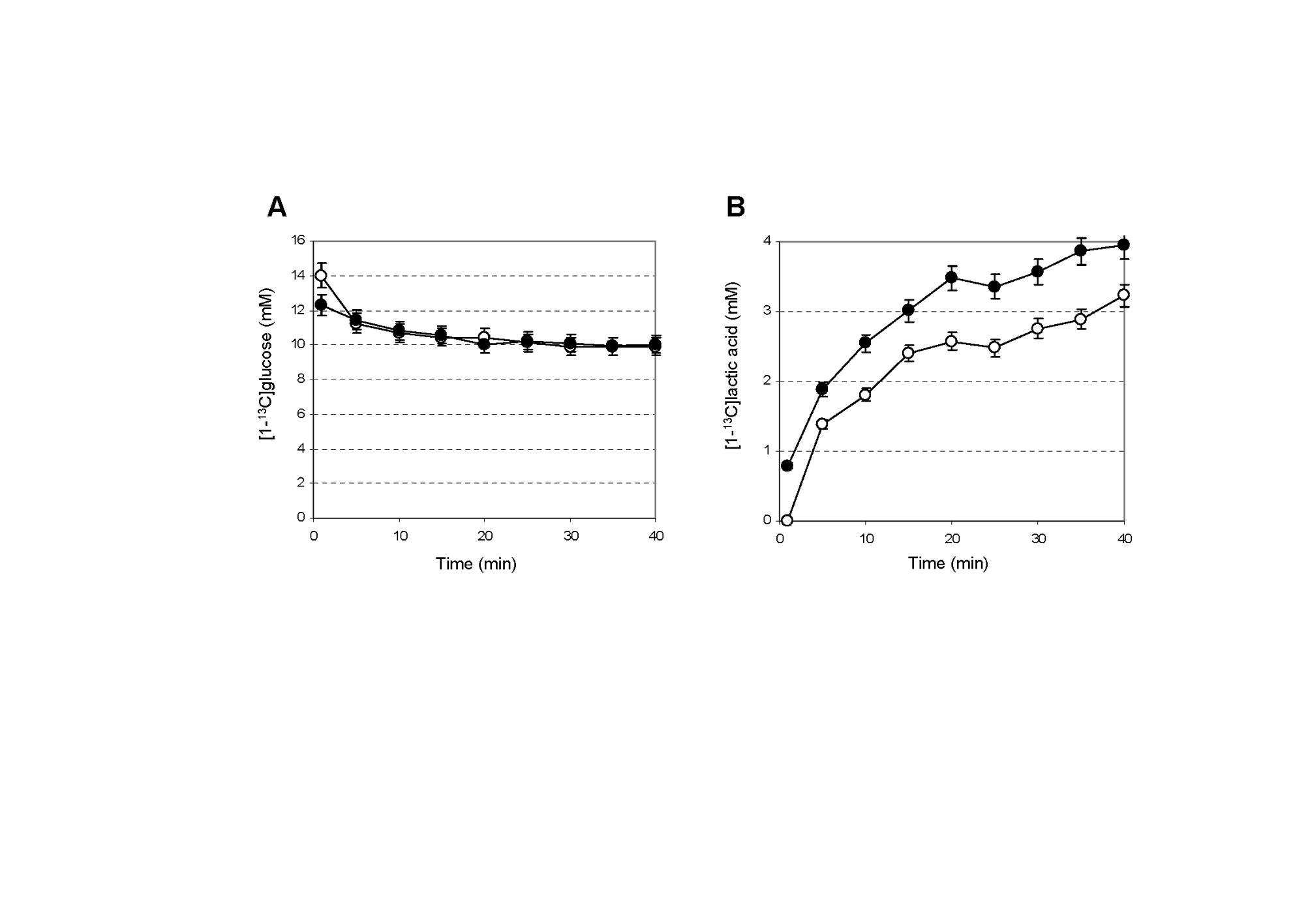

Supplement: Figure S2 — Time course of [1-13C]-glucose (14 mM). (A) and -lactic acid (B) consumption/product formation in L. lactis IL1403. The metabolite concentrations were measured in in vivo 13C NMR experiments using EdC that were activated with 14 mM lactose (white circles) or 14 mM lactose/1 mM ammonia (black circles). The error bars represent the SEM. (TIF) [file pone.0015520.s003.tif]

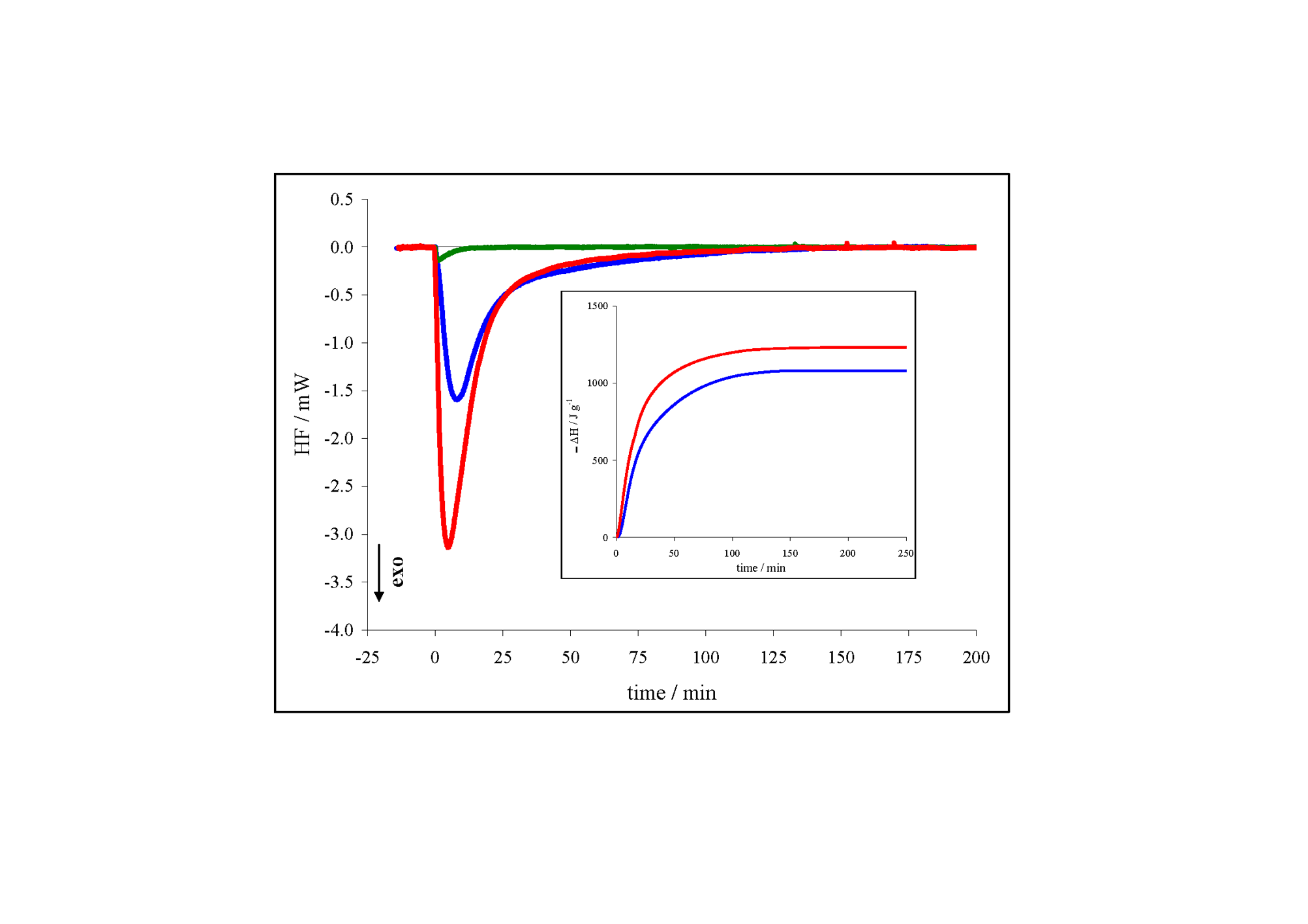

Supplement: Figure S3 — Raw isothermal titration calorimetry data (heat flux versus time) of L. lactis IL1403 lactose metabolism alone (blue line), or in the presence of ammonia (red line). The effect of ammonia dilution is also shown (green line). Lactose (70 mmol) or ammonia (5 mmol) was injected into a 5 ml suspension of EdC at time zero. The inset represents the overall specific enthalpy (with respect to grams of total protein) versus time. The details of the experimental conditions are provided in the supplementary materials. (TIF) [file pone.0015520.s004.tif]

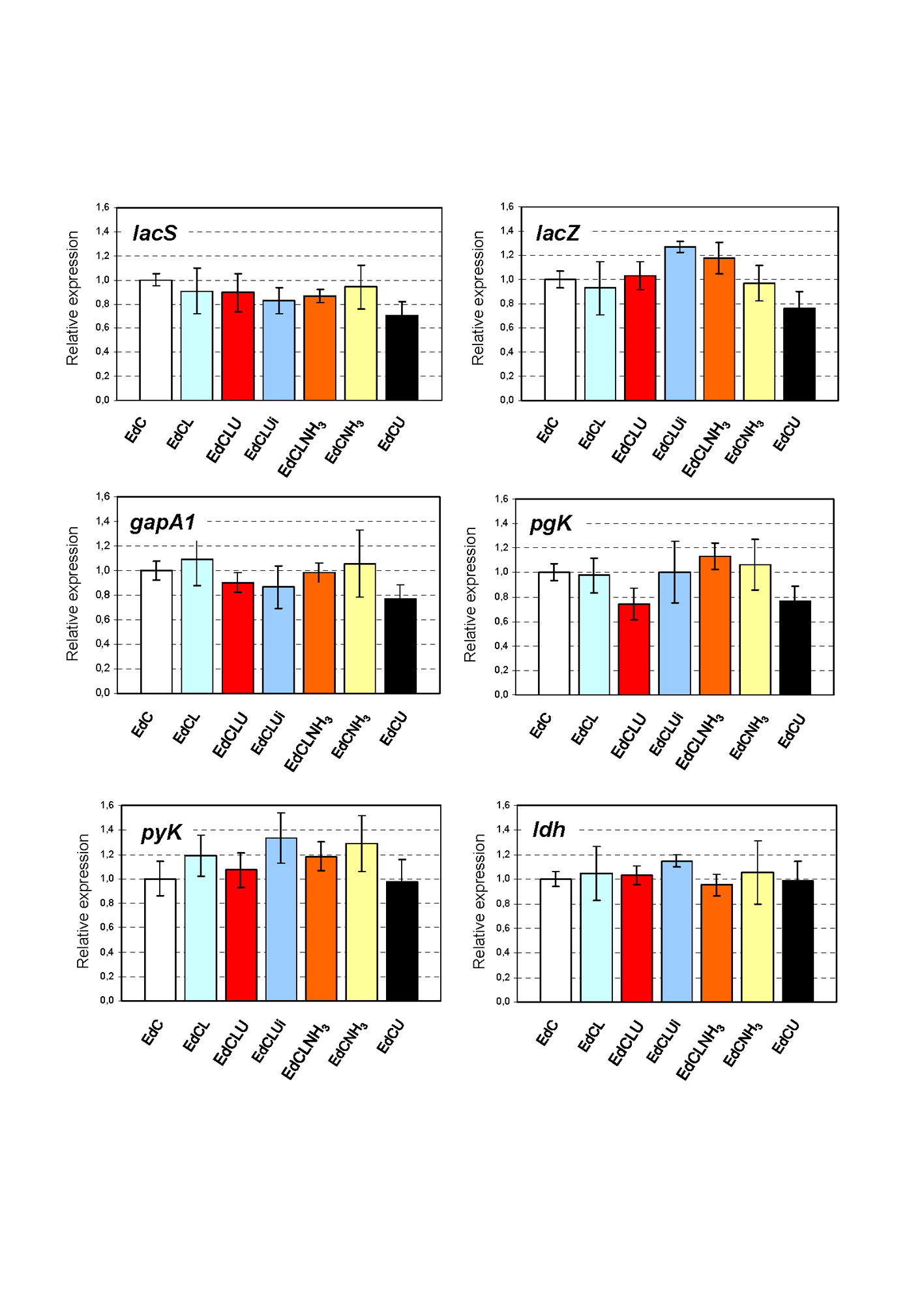

Supplement: Figure S4 — The relative expression of S. thermophilus genes involved in metabolism. lacS, lactose permease; lacZ, β-galactosidase; gapA1, glyceraldehyde-3-phosphatedehydrogenase; pgk, phosphoglycerate kinase; pyk, pyruvate kinase; ldh, lactate dehydrogenase. Total RNA was extracted from EdC, EdC activated with 14 mM lactose (EdCL), EdC activated with lactose and 1 mM urea (EdCLU), EdC activated with lactose and 1 mM ammonia (EdCLNH3) or EdC treated with NH3 (EdCNH3) or urea (EdCU). The expression levels of analyzed genes was normalized using polC, rpoC and murE as reference housekeeping genes. (TIF) [file pone.0015520.s005.tif]

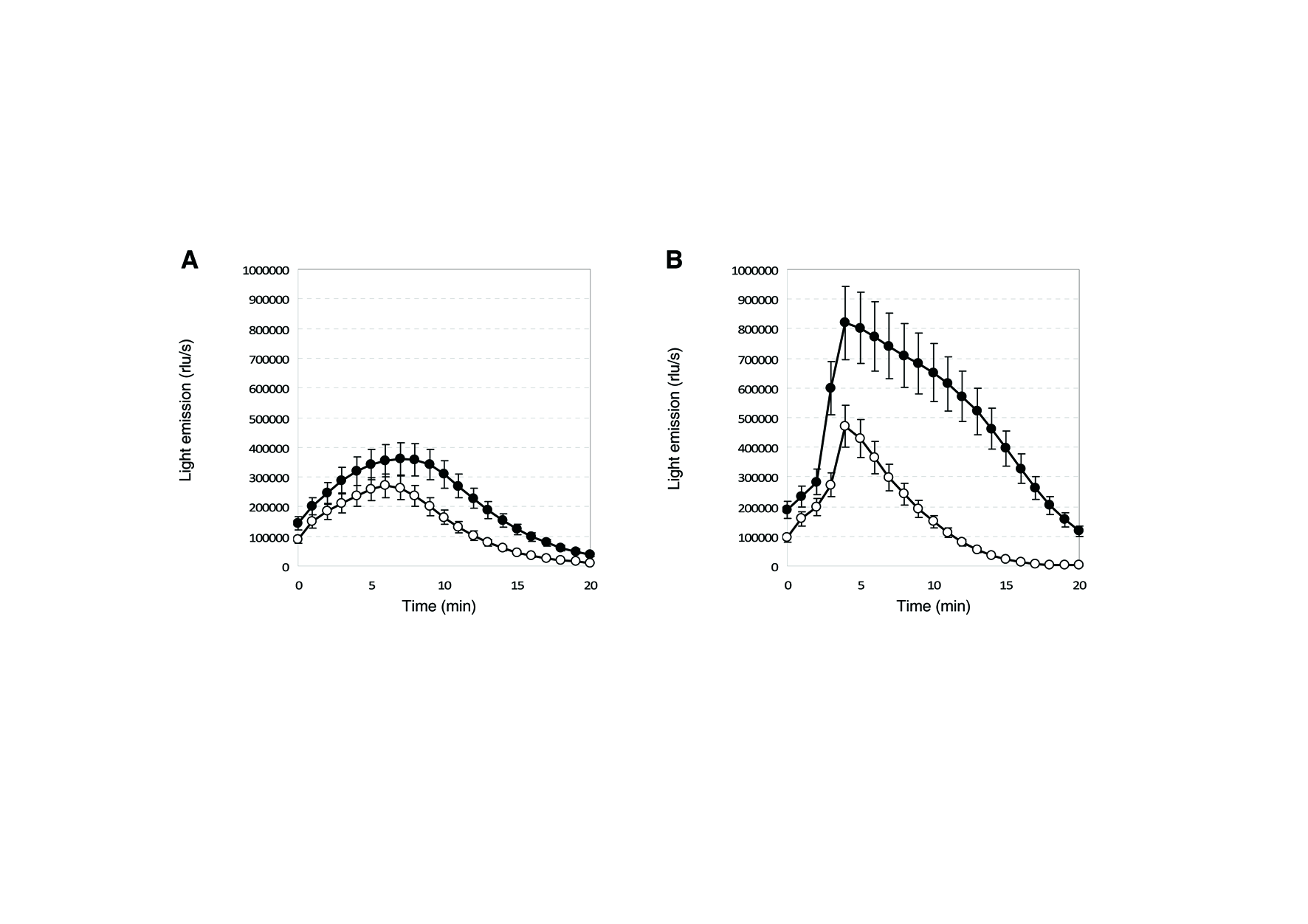

Supplement: Figure S5 — The intracellular ATP concentration presented as light emission, in S. pneumoniae FP292-945 EdC activated with 14 mM glucose (white circles) or 14 mM glucose and 1 mM ammonia (black circles) (A). The intracellular ATP concentration presented as light emission, in S. pneumoniae FP292-945 EdC activated with 14 mM cellobiose (white circles) or 14 mM cellobiose and 1 mM ammonia (black circles) (B). The error bars represent the SEM. (TIF) [file pone.0015520.s006.tif]
