# Supplementary material for: Alkalizing Reactions Streamline Cellular Metabolism in Acidogenic Microorganisms
Source: PLoS One. 2010 Nov 30;5(11):e15520. doi: 10.1371/journal.pone.0015520 (PMC2994868; doi:10.1371/journal.pone.0015520)
Supplement: Materials and Methods S1 — (DOC) [file pone.0015520.s007.doc]

**Supporting Materials and Methods**

**Bacterial strains, growth conditions, and reagents**

Wild-type *S. thermophilus* DSM 20617^T^ and its urease-negative mutant A16(*ΔureC3*) [1] were maintained in lactose M17 broth (20 g/l, lactose). Bioluminescent derivatives, MIM945 and A16-945, were maintained in M17 broth (Difco Laboratories, Detroit, MI) at 37 °C with 4 μg of chloramphenicol per ml. *Lactococcus lactis* IL1403 and its bioluminescent derivative IL1403-945 were maintained in M17 broth. Bioluminescent strains were obtained by electrotransformation of the *S. thermophilus* DSM 20617^T^, *S. pneumoniae* SP292 and *Lactococcus lactis* IL1403 with the shuttle vector pCSS945 containing the luciferase gene from *Pyrophorus plagiophtalamus* [2]. Electrotransformation of *S. thermophilus* and *L. lactis* was carried out as previously described [1]. Transformation of *S. pneumoniae* SP292 was performed following standard protocols for pneumococcal transformation as described by Iannelli [3]

**In Vivo NMR spectroscopy**

For ^13^C- and ^31^P-NMR experiments, *S. thermophilus* cells grown in M17 (20 g/l, lactose) were collected in late exponential grow phase (O.D. _600 nm_ 2.5), washed and suspended in solution A (9 g/l NaCl, 100 μg/ml chloramphenicol), containing 10 % (v/v) D_2_O, to reach a cell density correspondent to 8-9 mg of protein per ml. For evaluation of urea hydrolysis, cells suspension prepared as above were introduced in a standard 10-mm NMR tube. The sample was maintained at 37 °C and an initial spectrum was acquired. At a time designated zero, ^13^C-labelled urea (Sigma-Aldrich, Milano, Italy) was supplied at final concentration of 10 mM and a sequence of spectra were acquired during subsequent 2.5 min intervals. Pure dioxane in methanol, in a glass capillary, was used as the concentration standard. The quantitative kinetic data for urea were calculated from the area of the relevant resonance, in the sequence of in vivo spectra and comparing with the area of the resonance due to ^13^C-urea in solution A without cells.

The intracellular pH (pH_in_) was estimated from the pH dependence of the chemical shift of Pi in 50 mM MES buffer solution containing 5 mM MgCl_2_ and 85 mM NaCl as described by Neves et al. [4]. Extracellular pH was evaluated in the same condition described for the measurement of pH_in_ but using a standard pH Meter.

Lactose and glucose metabolism of non growing *S. thermophilus* and *Lactococcus lactis* were studied in energetically discharged cells. In specific, a cell suspension prepared in solution A as described before, was de-energized through an incubation at 37 °C (30 °C for *L. lactis*) for 20 min in presence of 10 mM urea (for urease-positive strain) or 5 mM ammonia (for urease-negative strain). After incubation the cell suspension was washed four times with solution A and called energetically discharged cells (EdC). The experiment were performed using a 5-mm NMR tube containing 600 μl of cell suspension. ^13^C NMR spectra were acquired sequentially before and after the addition of [1-^13^C]lactose or D-[1-^13^C]glucose (14 mM) and/or 1 mM urea and/or 1 mM ammonia. The labeled metabolites were monitored noninvasively for 40 min at 30 or 37 °C. The quantitative kinetic data for metabolites were calculated as described above for ^13^C-urea. All the NMR spectra were recorded with a Bruker AV-600 spectrometer, operating at a frequency of 600.10 MHz.

## Determination of ATP in whole cells, cell-free extract and membrane-free extract

## Cells grown in M17 (2% lactose) were collected in late exponential grow phase (O.D. _600 nm_ 2.5) or in stationary phase (O.D. _600 nm_ 4.0), washed and suspended in solution A (NaCl 9 g/l, 100 µg/l of chloroamphenicol) to give a cell density between 1-2 mg of protein per ml. Aliquots containing about 5 mg of dry weight of cells were incubated for 5 min at 37°C prior the addition of urea or NaHCO_3_ or NH_4_Cl at a final concentration of 5, 10 and 20 mM. When requested, the cell suspension was pre-incubated for 5 min at 37 °C in the presence of 10 μM flurofamide, or 20 µM gramicidine, or 200 µM m-chlorophenylhydrazone (CCCP), or 2 µM valinomycin 20 mM KCl, or 200 µM N, N-dicyclohexylcarbodiimide (DCCD). ATP synthesis was monitored during 4-15 min at 37 °C. ATP was extracted by rapidly boiling the cells suspension for 10 min. Cell debris were removed by centrifugation (20000 x g, for 15 min, 4 °C) and the ATP crude extract (ATPce) obtained was cooled on ice and used for ATP evaluation. Light emission was recorded 10, 30 and 50 s after the addition of 100 μL of ATP assay mix (SIGMA-Aldrich, Milano, Italy) to 100 μl of ATPce dilutions in a luminometer (Vega, Berthold Detection System, Germany); the ATP content was calculated after extrapolation to 0 s from a calibration curve with Na_2_-ATP as a standard. Intracellular concentration was calculated as described previously [5]. Crude Cell-free extracts (CE) were obtained as previously described [6] with the following modifications: total bacterial proteins were extracted from cells harvested by centrifugation from 200 ml of M17 culture grown at 37 °C for 12 h. Cells were washed twice in ATP buffer (20 mM Tris-Acetate, 1 mM EDTA, 100 μg/mL chloramphenicol, pH 7.75). Cell disruption was then carried out in a French Press (SLM Instrument, **Rochester, NY) and the resulting cellular extract was centrifuged at 20000 x g for 30 min. The protein content of the supernatant was determined using the Bradford method [7] and about 2-4 mg of proteins were used for the evaluation of ATP synthesis in several conditions in presence of urea and/or other chemicals. Membrane-free extract (MfE) was obtained centrifuging the cell-free extract at 100.000 x g for 30 min and collecting the supernatant. Cell-free and membrane-free extract with and without the addition of triton X100 (0,5%) were used for ATP determination as described above for cell suspensions. When requested, the enzymatic activity present in CE and MfE were subjected to a thermal inactivation performed at 100 °C for 5 min.**

**Light emission measurement**

Light emission from *S. thermophilus* and *Lactococcus lactis* strains harboring the pCSS945 vector [2] is directly correlate with the ATP pool because the enzymatic reaction catalyzed by the click beetle (*Pyrophorus plagiophthalamus*) enzyme is the follow: luciferase ATP + D-luciferin +O_2_ → AMP + oxyluciferin + PP_i_ + CO_2_ + light (~560 nm). Light emission was measured in solution A supplemented with D-luciferin at a final concentration of 0.2 mM. D-luciferin was prepared in solution A. In specific, EdC prepared as described for in vivo NMR spectroscopy, was dispensed (100 μl) in a 96 wells microplate and light emission was measured in a Victor3 Luminometer (PerkinElmer, Monza, Italy) programmed for a reading set of 20 repetitions with intervals of 1 min at 37 °C. The light emission from recombinant *S. thermophilus*, *S. pneumoniae* and *Lactococcus lactis* strains was measured in absence or in presence of different combination of the following chemicals, 14 mM lactose, 14 mM glucose, 14 mM cellobiose, 1 mM urea, 1-2 mM ammonia, 0.1 M sodium oxamate (Sigma-Aldrich, Milano, Italy).

**Intracellualr pH measurement**

The internal pH of *S. thermophilus* DSM 20617^T^ was measured at external-pH values of 6.0 to 9.0, using the pH-sensitive fluorescence probe 5 (and 6-)-carboxyfluorescein succinimidyl ester (cFSE), as described by Sawatari and Yokota [8] based on the method originally described by Breeuwer [9], with some modification. The fluorescence intensity of this probe is increased at **alkali**ne pH and reduced at acidic pH. A *S. thermophilus* cell suspension in solution A obtained as described before was diluted to an OD_660nm_ of around 0.5 and supplemented with 4 µM cFDASE, a precursor molecule of cFSE, incubated for 30 min at 37 °C. During this incubation, the membrane-permeating cFDASE was cleaved by intracellular esterases, and the resultant cFSE molecules were conjugated to the aliphatic amines of intracellular proteins. After being centrifugated and washed with solution A, the cells were resuspended in the same volume of solution A. The nonconjugated probe was eliminated by the addition of glucose at a final concentration of 14 mM and subsequent incubation for 1 h at 37°C. After being washed and resuspended in solution A, their energy was depleted through incubation for 1 h at 37 °C. After being centrifuged and washed with solution A, the cells were resuspended to reach a final cell concentration corresponding to 7-8 mg proteins per ml. The cells suspension was dispensed (100 μl) in a 96 wells microplate and the internal pH was determined by measuring the fluorescence intensities of the cell suspension with excitation and emission wavelengths of 490 and 520 nm respectively, using a fluorimeter/luminometer Victor 3 programmed for a reading set of 20 repetitions with intervals of 30 s at 37 °C. The change in pH_in_ was measured in cells energized with different combination of 14 mM lactose, 0.5 mM urea, 1 mM ammonia and 0.4 M sodium oxamate. An aliquot of the same cell suspension was washed and resuspended in different buffers with a pH ranging form 5 to 11.5, treated with 100 μM gramicidine, which dissipates the transmembrane proton gradient, and the fluorescence intensity was measured for calibration at appropriate external-pH values adjusted with the corresponding **alkali** (NaOH or KOH) and HCl.

**Measurement of pHì-dependent glycolysis and homolactic fermentation activity**

To evaluate the pH dependency of *S. thermophilus* glycolysis and homolactic fermentation activity, the rate of glucose consumption and lactic acid production were measured in the presence of 100 μM gramicidine at various pHs between 5.0 and 11.5. The cells were collected in late exponential grow phase (O.D. _600 nm_ 2.5) in M17 medium, harvested, and washed with solution A. The washed cell pellet was resuspended in the same solution and incubated for 1 h at 37 °C to deplete their metabolic energy. The cells were concentrated to obtain a cell density corresponding to 7-8 mg of protein per ml in one of the following buffers at 100 mM in presence of 100 μM gramicidine with the pH adjusted to the desired values, using KOH and HCl: MES (pH 4.0 to 7.0), Tris-HCl (pH 7.5-11.5). The reaction was started by the addition of glucose at a final concentration of 14 mM. The mixtures were incubated at 37 °C for 1 h. After being incubated, the mixtures were centrifuged at 18,120 x g for 5 min at 4°C, and the supernatant was filtered through a 0.2-µm-pore-size membrane. The glucose and lactic acid concentrations of the filtered supernatants were measured by HPLC analysis. The separation of glucose and lactic acid was performed by means of HPLC using a Waters (Milford, MA) equipment consisting of an Alliance 2695 pump system and a 410 refractive index detector. A Bio-Rad Aminex HPX 87H column (300 x 7.8 mm, Bio-Rad Laboratories, Hercules, CA) maintained at 50 °C was used. The isocratic elution was run at a flow rate of 0.6 ml/min using 0.01 N phosphoric acid aqueous solution as mobile phase. Data were collected and processed using Millennium™ software (Waters). Glucose and lactic acid concentrations were calculated by the external standard method using aqueous standard solutions of glucose and lactic acid (Sigma, St. Louis, MO, USA). All analyses were run in triplicate.

**Assay of β-galactosidase and lactate dehydrogenase activities**

β-galactosidase activity of *S. thermophilus* wild-type and A16(*ΔureC3*) urease-negative mutant, was carried out on permeabilised cells obtained as described by Krishnan [10] with the following modification. The cells were collected in late exponential grow phase (O.D. _600 nm_ 2.5), washed and suspended in solution A and an equal volume of 10% (v/v in water) toluene was added. The suspension was incubated for 2 min at 30 °C with shaking (100 rpm, Unimax 2010, Heidolph Instruments, Milano, Italy). The permeabilised cells were recovered by centrifugation (12,000 x g, 5 min), washed twice in solution A and concentrated to reach a cell density correspondent to 8-9 mg of protein per ml and used immediately for β-galactosidase assay. Measurement of the β-galactosidase activity was performed in 200 μl aliquots of permeabilised cell suspension containing 0.2 mg/ml of 2-nitrophenyl-β-D-galactopyranoside (Sigma-Aldrich, Milano, Italy) at 37 °C, by monitoring the optical density at 420 nm with a microplate-reader M680 (Bio-Rad Laboratories, Hercules, CA, USA) programmed for a reading set of 60 repetitions with intervals of 30 s. The β-galactosidase activity was expressed in mO.D._420 nm_ per min, as the mean of four independent determinations. The assay was performed in presence of different concentration of urea (0.1-20 mM) or ammonia (0,2-40 mM). Lactate dehydrogenase activity was determined by measuring the rate of NADH oxidation at 340 nm (380 nm for NADH concentration above 0,3 mM; ε = 1.244 l/mmol cm) essentially as described by Gaspar [11]. In specific, cells collected in late exponential growth phase washed in 100 mM Tris-HCl buffer pH 7 and concentrated were subjected to a mechanical disruption using a Precellys bead beater (Advanced Biotech Italia srl, Seveso, Italy). The amount of total proteins of each lysate was measured using the Bradford method with BSA as the standard. The pH profiles of lactate dehydrogenase activity were evaluated in assay mixture containing 0,3 mM NADH, 20 mM pyruvate, 5 mM MgCl_2_ in 50 mM KPi or 100 mM Mes/KOH for pH ranging from 5-6,5 and Tris-HCl 100 mM for pH ranging from 7 to 9. The lactate dehydrogenase activity was expressed as the mean of four independent determinations.

**Isothermal Titration Calorimetry (ITC)**

The influence of urea and/or ammonia to the cell bioenergetics of the microorganisms was monitored by means of ITC. The instrument used was the “Calorimètre E. Calvet pour Microcalormétrie, DAM” (Setaram, Lyon, France) equipped with 10 cm^3^ volume capacity stainless steel measurement compartment and the injection system Microlab 500 (Hamilton Company, Reno, RV). Calibrations was performed using the Joule effect calibrator EJ2 (Setaram). 5 ml of *S. thermophilus* DSM20617^T^ and *L. lactis* IL1403 EdC were loaded in the measurement compartment. Measurements where performed at 30 °C and 37 °C with a 70 rpm stirring applied. After 2h equilibration, 70 μL of lactose, lactose plus urea or lactose plus ammonia solution was injected. The concentration of the solutions where chosen in order to obtain 14 mM lactose, 0.5 mM urea, 1 mM ammonia as final concentrations.

The Heat Flux versus time row signal was numerically integrated to obtain the overall specific enthalpy (with respect of grams of total cells protein amount) ΔH/J g. The dilution heat effects and the heat effect of urea hydrolysis were taken into account (subtracted from the overall heat variation measured). Three replicates were performed for each experiments. The ΔH errors was minor to 4%.

**Transcriptional analysis of genes involved in homolactic fermentation**

Total RNA was extracted from EdC or EdC activated with 14 mM lactose (EdCL), or EdC activated with lactose and 1 mM urea (EdCLU), or EdC activated with lactose and 1 mM ammonia (EdCLNH_3_) or EdC treated with NH3 (EdCNH_3_) or urea (EdCU). A volume of EdC (corresponding to 6 mg of dry weight cells) was collected after 5 min of activation at 37 °C, centrifuged (4 °C , 1 min at 8000 g) and resuspended in 1 ml of PureZol (Bio-Rad Laboratoires, Milano, Italy) and subjected to a mechanical disruption using a Precellys bead beater (Advanced Biotech Italia srl, Seveso Italy). Total RNA extraction was continued following the extraction procedure of Aurum Total RNA Fatty and Fibrous Kit (BioRad Laboratoires, Milano, Italy). 1 μg of RNA was used for cDNA synthesis using iScript^TM^ cDNA Synthesis (BioRad, Laboratoires, Milano, Italy). RT-qPCR was carried out using 5 μl of cDNA solution in a total volume of 20 μL using the EvaGreen^TM^ kit (BioRad, Laboratoires, Milano, Italy). The expression levels of *lacS,* lactose permease, *lacZ,* β-galactosidase, *gapA1*, glyceraldehyde-3-phosphatedehydrogenase, *pgk*, phosphoglycerate kinase, *pyk*, pyruvate kinase, and *ldh*, lactate dehydrogenase were normalized using *polC*, *rpoC* and *murE* as reference housekeeping genes. For each condition, the measures triplicated with cDNA synthesized from two independent RNA samples. The real-time PCR was carried out using the EvaGreen^TM^ PCR master mix (BioRad, Laboratoires, Milano, Italy) as recommended. PCRs were performed in triplicate and run on the CFX96 (BioRad, Laboratoires, Milano, Italy). Data were recorded as threshold cycles (*C_T_*), expressed as means ± standard deviations, computed using the software BioRad CFX Manager^TM^ and expressed as normalized expression (ΔΔ*C_T_*) ± standard error of the mean.

The primer sets used in real-time PCR experiments are shown in Table 1.

Table 1. Real-time PCR primers sequences

| Gene target | Primers |
| --- | --- |
| *lacS* | lacS1f (GGCAGGAATCATTGACCAGAAACC)  lacS1r (ATCACCTTGTTGCTTCTCTTCACC) |
| *lacZ* | lacZf (TTGACCACGAACAATTTCATGACC)  lacZr (ACCAGGCGAAGAAGCAACATTC) |
| *gapA1* | gapA1f (ggaactggaacacgttgtgcagat)  gapA1r (ggtgaccaaatgatccttgacgga) |
| *pgK* | pgkf (gttaccgatttcgataccttgcgc)  pgkr (atagccacgaatggacgttctgga) |
| *Pyk* | pykf (tacttcagaacgagttgcacgtgg)  pykr (acttcgatacccatgtcaccacga) |
| *Ldh* | ldhf (TAGTTATCACTGCTGGTGCTCCTC)  ldhr (TCTACTGGGTTAGCAGCTACAAGG) |
| *polC* | polCf (TTTGATGCCCGAGGGACAGAAG)  polCr (CTGTTATCGCAACTGCTGGATGTC) |
| *rpoB* | rpoBf (ACCCAAGTTACGAGCAGCCATAC)  rpoBr (TTCCAGACGGTACACCAGTTGAC) |
| *murE* | murEf (TCAATAGGTCCGATGTGGTCTGG)  murEr (CCGTATGATGGCTGAAGCTGTATC) |

All the primers were designed to work at an annealing temperature of 61 °C. The optimal annealing temperature was tested for all the primer sets using a temperature gradient PCR protocol ranging from 57 to 72 °C. The amplification efficiency (%), tested using serial dilution of quantified DNA extracted from *S. thermophilus* DSMZ 20617^T^, was between 95 and 100% for all the primer sets.

**References**

1. Mora D et al. (2004) Characterization of urease genes cluster of *Streptococcus thermophilus*. *J Appl Microbiol* 96:209-219.
2. Loimaranta V, Tenovuo J, Koivisto L, Karp M (1998) Generation of bioluminescent *Streptococcus mutans* and its usage in rapid analysis of the efficacy of antimicrobial compounds. *Antimicrob Agents Chemother* 42:1906-1910.
3. Iannelli F, Pozzi G (2004) Method for introducing specific and unmarked mutations into the chromosome of *Streptococcus pneumoniae*. *Mol Biotechnol* 26:81-86.
4. Neves, AR et al. (2004) In vivo nuclear magnetic resonance studies of glycolitic kinetics in *Lactococcus lactis*. *Biotech Bioeng* 64:200-212.
5. Palmfeldt J, Paese M, Hahn-Haegerdal B, van Niel EWJ (2004) The pool of ADP and ATP regulates anaerobic product formation in resting cells of *Lactococcus lactis*. *Appl Environ Microbiol* 70:5477-5484.
6. Mora D et al. (2005) Urease biogenesis in *Streptococcus thermophilus*. *Res Microbiol* 156:897-903.
7. Bradford MM (1976) A Rapid and Sensitive Method for the Quantitation of Microgram Quantities of Protein Utilizing the Principle of Protein-Dye Binding. *Meth Biochem*, 72:248-254.
8. Sawatari Y, Yokota A (2007) Diversity and mechanisms of alkali tolerance in Lactobacilli. *Appl Environ Microbiol* 73:3909-3915.
9. Breeuwer P, Drocourt JL, Rombouts FM, Abee T (1996) A novel method for continuous determination of the intracellular pH in bacteria with the internally conjugated fluorescent probe 5 (and 6-)-carboxyfluorescein succinimidyl ester. *Appl Environ Microbiol* 62:178-183.
10. Krishnan S, Gowda LR, Karanth NG (2000) Studies on lactate dehydrogenase of *Lactobacillus plantarum* spp. involved in lactic acid biosynthesis using permeabilised cells. *Proc Biochem* 35:1191–1198.
11. Gaspar P et al. (2007) The lactate dehydrogenases encoded by the *ldh* and *ldhB* genes in *Lactococcus lactis* exibit distinct regulation and catalytic properties – comparative modelling to probe the molecular basis. *FEBS J.* 274:5924-5936.
